# Commentary: Descriptions of Disordered Eating in German Psychiatric Textbooks, 1803 – 2017

**DOI:** 10.3389/fpsyt.2021.669387

**Published:** 2021-04-12

**Authors:** Martin Brüne

**Affiliations:** Division of Social Neuropsychiatry and Evolutionary Medicine, Department of Psychiatry, Psychotherapy and Preventive Medicine, LWL University Hospital, Ruhr University Bochum, Bochum, Germany

**Keywords:** eating disorders, anorexia nervosa, psychiatry, psychosomatic medicine, history

Bergner et al. thankfully summarize the historical reception of the concept of eating disorders, with a focus on what is nowadays referred to as “anorexia nervosa” (1). They point out that, despite the first scientific descriptions of the syndrome by William Gull (1868/1873 in England) and Ernest-Charles Lasègue (1873 in France) (2, 3), the first entry in a German Textbook of Psychiatry was traced to occur as late as 1974. In fact, the authors assert that early twentieth century psychiatrists including Emil Kraepelin, Oswald Bumke, and Eugen Bleuler, to name just a few, were apparently oblivious of the disorder, and that Gerd Huber, chairman of psychiatry at the University of Bonn, was the first to mention it in his textbook as “anorexia mentalis” (4).

Here, I would like to argue that the authors are incorrect in this point. Gerd Huber (1921-2012) was not just known for the description of coenesthetic schizophrenia, he also promoted pneumencephalography as a method to demonstrate enlargement of the lateral ventricles in people diagnosed with schizophrenia. Huber, as far as is known to this author, was never an expert or had a special interest in eating disorders.

What Bergner et al. apparently overlooked is a monograph entitled “Anorexia nervosa,” authored by Helmut Thomä (1921-2013), which was published in 1961 (5). The book is based on Thomä's habilitation thesis (which is similar to the degree of a Ph.D.) comprising numerous case reports and detailed descriptions of therapeutic interventions as well as psychological mechanisms putatively involved in the etiology of anorexia nervosa. Thomä had a strong background in psychoanalysis and, in 1962, received a call to become chairman in Psychosomatic Medicine and Psychoanalysis at the University of Ulm. In retrospect, this event was a hallmark, as Thomä was the first to hold a chair in Psychosomatic Medicine in Germany, thus marking the separation of Psychosomatic Medicine from Psychiatry that Bergner et al. describe so nicely.

In the first chapter of his monograph, Thomä accurately illustrates the historical development of anorexia nervosa as a “disease entity.” In fact, Gull initially called the condition “apepsia hysterica,” and later suggested to rename it in the way we use the term until the present day. More importantly, Thomä quotes Otto Soltmann, a German pediatrician, who, in 1894, explicitly referred to the work of Gull and Lasègue (6). Soltmann called the syndrome “anorexia cerebralis.” Similarly, A. Kissel, another German pediatrician described a case of an 11-year old girl suffering from anorexia nervosa in 1896 (7). Also, Hans Curschmann (better known for his clinical description of myotonia dystrophica, together with Hans Steinert), a German internist, was well aware of the syndrome of anorexia nervosa, particularly with regard to its differentiation from other diseases associated with pituitary dysfunction (8). Indeed, as Bergner et al. rightly point out, throughout the first half of the twentieth century, interest in anorexia nervosa was greater in medical disciplines other than psychiatry than in psychiatry itself (of note, the German psychiatrist Heinrich Hoffmann (1809-1894), who was the clinical director of the psychiatric hospital in Frankfurt/Main, published, in 1844, a children's book on the occasion of his son's birthday, entitled “Der Struwwelpeter” (9). Herein, Hoffmann gave a detailed description of a male (!) case of anorexia nervosa (with a fatal outcome), aside from case descriptions of other psychiatric conditions that are nowadays apparently much more prevalent than in the nineteenth century, including attention deficit/hyperactivity disorder).

However, although I did not carry out a systematic survey of the literature, it is noteworthy that anorexia nervosa was explicitly referred to by Gottfried Ewald (1888-1963), Professor of Psychiatry at the University of Göttingen, in his textbook “Neurologie und Psychiatrie” (5th edition, 1964) (10). Interestingly, Ewald listed anorexia nervosa among the diseases of the autonomic nervous system, specifically endocrinological disorders (page 202 ff.). It is unknown to me, however, whether or not the description of anorexia nervosa was already part of the first edition of Ewald's textbook published in 1944.

Aside from Ewald's textbook of Neurology and Psychiatry, “anorexia mentalis” appeared in a textbook entitled “Psychosomatische Medizin” (i.e., “Psychosomatic Medicine”) published by the Swiss psychiatrist Medard Boss (1903-1990) in 1954 from the perspective of “Daseinsanalyse” (“analysis of existence”) (11). Back then, analysis of existence was a wide-spread concept in psychiatry (and psychosomatic medicine), essentially promoted by Ludwig Binswanger (1881-1966) and others to improve the understanding of psychological conditions as they unfold in the inner and outer world. As an interesting side-note, Boss worked at the famous Burghölzli in Zurich under the supervision of Eugen Bleuler, such that it is very unlikely that Bleuler was unaware of the syndrome of anorexia nervosa, even though he did not seem to mention it in his textbook (though later acknowledged by his son, Manfred Bleuler who edited several subsequent editions).

As Bergner et al. rightly emphasize from a historical perspective, around the turn of the nineteenth century, cases of anorexia nervosa were hidden behind categories of mental disorders that are no longer in use, including “neurasthenia,” “hysteria,” and “hypochondria.” Before this background, it may be worth mentioning that Theodor Ziehen (1904-1912), from 1904 to 1912 chairman at the Charité in Berlin, used (or introduced) the term “neurasthenische Anorexie” (i.e., “neurasthenic anorexia”) in his textbook of psychiatry as early as 1911, however, without referring to the typical clinical picture mainly observed in adolescent or young adult females [(12), p. 581].

In summary, while Bergner et al. are correct in their assumption that the concept of eating disorders was scarcely mentioned in German textbooks of psychiatry, their assumption that the term “anorexia nervosa” first occurred in 1974 in a German textbook is wrong. Rather, it seems that the concept was well-known long before among pediatricians and people who, especially after World War II, founded Psychosomatic Medicine and cemented the split of this medical discipline from psychiatry.

## Author Contributions

The author confirms being the sole contributor of this work and has approved it for publication.

## Conflict of Interest

The author declares that the research was conducted in the absence of any commercial or financial relationships that could be construed as a potential conflict of interest. The handling editor declared a shared affiliation, though no other collaboration, with the author MB.
